# Effects of flaxseed and *Hypericum perforatum* on hot flash, vaginal atrophy and estrogen-dependent cancers in menopausal women: a systematic review and meta-analysis

**Published:** 2016

**Authors:** Masumeh Ghazanfarpour, Ramin Sadeghi, Robab Latifnejad Roudsari, Talat Khadivzadeh, Imaneh khorsand, Maliheh Afiat, Mahdi Esmaeilizadeh

**Affiliations:** 1 *Student Research Committee, Department of Midwifery and Reproductive Health, Nursing and Midwifery School, Mashhad University of Medical Science, Mashhad, Iran*; 2 *Nuclear Medicine Research Center, Mashhad University of Medical Sciences, Mashhad, Iran*; 3 *Evidence-Based Care Research Centre, Department of Midwifery, School of Nursing and Midwifery, Mashhad University of Medical Sciences, Mashhad, Iran*; 4 *Evidence-Based Care Research Centre, Department of Midwifery, School of Nursing and Midwifery, Mashhad University of Medical Sciences, Mashhad, Iran*; 5 *Department of Microbiology, Islamic Azad University of Varamin-pishva,Tehran, Iran*; 6 *Obstetrics and Gynecology, Women's Health Research Center, Department of Obstetrics and Gynecology, Faculty of Medicine, Mashhad University of Medical Sciences, Mashhad, Iran*; 7 *Esfarayen faculty of Medical Sciences, Esfarayen, Iran*

**Keywords:** *Hot flash*, *Vaginal atrophy*, *Menopause*, *Systematic review*, *Cancers*

## Abstract

**Objective:**

In this study, we aimed at evaluation of the efficacy of *Hypericum perforatum* and flaxseed on hot flash, vaginal atrophy and estrogen-dependent cancers in menopausal women

**Materials and Methods:**

We searched MEDLINE, Scopus, and the Cochrane Central Register of Controlled Trials (RCT) to explore trials that assessed the effectiveness of *H. perforatum* and flaxseed on hot flash, vaginal atrophy and estrogen-dependent cancers. In this regard, the following terms were used “menopause AND *H. perforatum* OR flaxseed OR *Linum usitatissimum*. Only randomized controlled trials were included in the study.

**Results:**

Nine RCTs were included in this systematic review. Based on the literature, flaxseed showed beneficial effect on hot flash frequency and intensity, which was not statistically significant. According to two trials, flaxseed showed estrogenic effects; however, no conclusion regarding cancer promoting or protecting effects can be made. The evidence of the efficacy of the flaxseed on alleviating vaginal atrophy was also limited due to inconsistent findings in this regard. One trial declared that *Vitex agnus-castus* and *H. perforatum* showed comparable decrease in the frequency of hot flashes.

**Conclusion:**

The results of our systematic review suggest beneficial effect on vasomotor symptom with both of flaxseed and *H. perforatum*. Consistent conclusion regarding estrogen-dependent cancers and maturation value is limited due to small number of trials related to flaxseed. Further trials are still needed to confirm the results of our systematic review.

## Introduction

Menopause is a stage that all women experience in their life time (Ghaderi et al., 2010 ▶). Menopause happens around the age of 50 and is diagnosed by at least 12 months of amenorrhea. Menopause is often accompanied by a clustering of symptoms, including hot flashes, night sweat, vaginal atrophy, anxiety, nervousness and reduced libido (Cramer et al., 2012 ▶). Hormone therapy (HT) has been widely used in menopausal women seeking alleviation of symptoms of menopause (MacLennan et al., 2004 ▶, Loprinzi et al., 2000 ▶, Hidalgo et al., 2005 ▶). However, after publishing the first finding of the Women’s Health Initiative study, concerns was outlined about increased breast cancer risk (Group, 1998). Fear of breast cancer has driven users away from HT and consequently, there has been a sharp decline in the use of HT. Increasingly, many of the previous users of HT are getting interested in the flaxseed, *Vitex agnus-castusand* and *Hypericum perforatum* (*H. perforatum*) as alternatives to HT in the management of menopausal symptoms (Uebelhack et al., 2006 ▶, van Die et al., 2009 ▶, Ghazanfarpour et al., 2013 ▶, Dodin et al., 2005a ▶, Geller et al., 2009 ▶). Indeed, flaxseed is the richest food source of lignans, one of the three major classes of phytoestrogen (Geller et al., 2009 ▶). The aim of this systematic review was to assess the effect of flaxseed and *H. perforatum* on hot flashes, vaginal atrophy and estrogen-dependent cancers. 

## Material and Methods

We searched MEDLINE, Scopus, and the Cochrane Central Register of Controlled Trials to explore trials that assessed the effect of *H. perforatum* and flaxseed on hot flash, vaginal atrophy and estrogen-dependent cancers. No language restriction was imposed. Here, we searched the terms “menopause AND *Hypericum perforatum* OR flaxseed OR *Linum usitatissimum*”. In addition, reference sections of the relevant trials, systematic review and meta-analysis were manually checked to identify further trials missed by electronic search and the authors were contacted by email to obtain additional data. Also, sensitivity analysis was performed to assess the influence of excluding each study on heterogeneity.

**Inclusion criteria**

The inclusion criteria were limited to randomized controlled trials that used flaxseed or *H. perforatum* alone or in combination with other herbal medicine.

**Important data extraction**

Data was independently assessed by two authors and disparities were resolved by discussion with a third researcher. We estimated the differences in means in two ways: difference in means (MD) and standardized difference in means (SMD). The latter was used when studies included in the meta-analysis measured the same outcome by different measurement units. In the included studies, changes in mean at baseline and endpoint were assessed. Average and SD of differences between pre and post-trial variables were extracted, if possible. In other studies, pre and post-trial average and SD of the evaluated variables were extracted. Standardized or non-standardized difference in means was calculated for each study as the effect sizes.

**Statistical analyses**

We interpreted the results using random effects model (Der-Simonian and Laird method) because of the presence of large heterogeneity among included trials in our meta-analysis. For heterogeneity evaluation, Cochrane Q test (p<0.05 as statistically significant) and I^2 ^index were used. I^2^ index was use to assess how much of the variance across studies is likely to be real and is not due to sampling errors. All statistical analyses were done by Comprehensive Meta-analysis Version 2 (Biostat, Englewood, NJ, USA). We also included data only from first phase of Baber (Baber et al., 1999 ▶) and Lipovac trials (Lipovac et al., 2012 ▶) to main meta-analysis in order to avoid the carry-over effect. 

## Results

**Figure 1:**
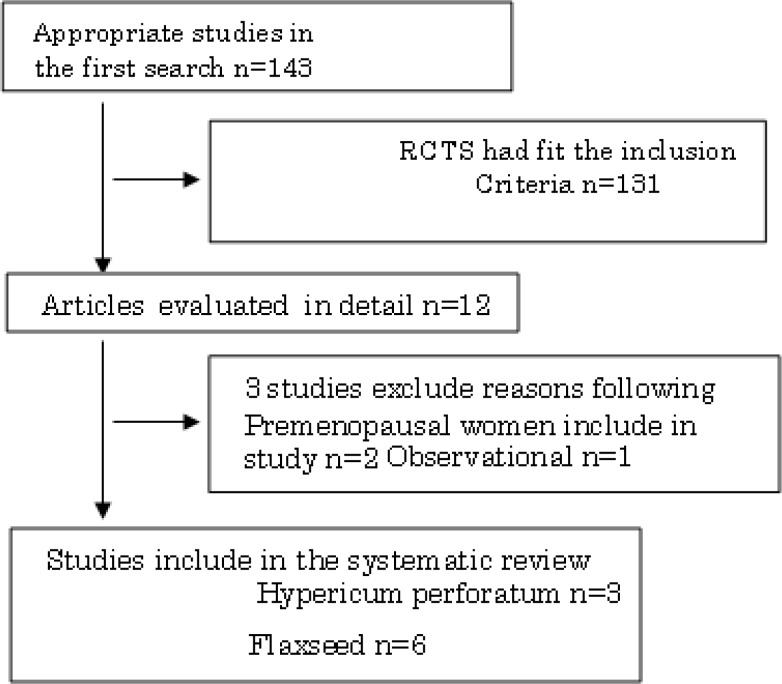
Search strategy of our study

Out of a total of 143 studies, which were found in the first search, 131 studies were not Randomized Controlled Trials (RCTs) and were excluded from our systematic review. The remaining 12 articles were assessed in detail. Finally, 9 RCTs met the inclusion criteria. Table 1 shows the characteristics of the nine selected RCTs. The process of the search and selection of RCTs is shown in Figure 1.

**The effect of flaxseed on the severity of hot flashes**

**Figure.2:**
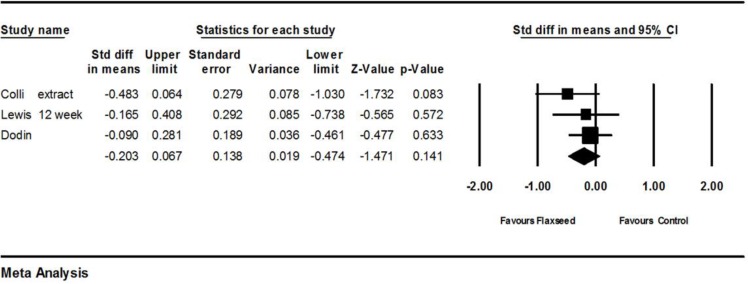
Effects of flaxseed on severity of hot flashes, the horizontal lines denote the 95% CI, ■ point estimate (size of the square combined overall effect of treatment

Three studies (Colli et al., 2012 ▶; Lewis et al., 2006a ▶; Dodin et al., 2005b ▶) provided complete statistical data for the meta-analysis. Standardized difference in means (SMD) of the severity of hot flash in flaxseed group was lower as compared to control group (–0.203; 95%CI: -0.474 to 0.067; p=0.141; heterogeneity p=0.501; I^2^=0%; 212 participants; random effects model). The forest plot is shown in Figure 2. Coli et al. compared 3 arms (1 g of flaxseed extract containing 100 mg lignan, 90 g flaxseed meal containing 270 mg lignan and placebo) (Colli et al., 2012 ▶). Inclusion of flaxseed extract arm only into the meta-analysis showed no significant change in the pooled effect size as compared to the main analysis. On the other hand, by inclusion of flaxseed meal arm only, pooled effect size showed a less decrease in hot flash severity as compared to the main analysis (SMD=-0.163, 95%CI: -0.437 to 0.111; p=0.245). 

**The effect of flaxseed on the frequency of hot flashes**

**Figure 3:**
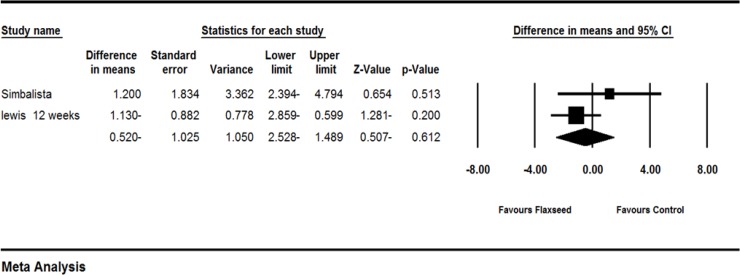
Effects of flaxseed on the frequency of hot flashes, the horizontal lines denote the 95% CI, ■ point estimate (size of the square corresponds to its weight); ♦, combined overall effect of treatment

Two studies (Simbalista et al., 2010 ▶; Lewis et al., 2006a ▶) provided sufficient statistical data to be included in the meta-analysis. Difference in means (MD) of frequency of hot flash showed greater decrease in flaxseed group as compared to the control group (MD -0.520[-2.528 to 1.489]; p= 0.150 I^2^=0%; random effects model). The forest plot is shown in Figure 3. FDA and the European Medicines Agency (EMEA) have considered at least 2 hot flash/day as clinically meaningful (Colli et al., 2012 ▶). The results of our meta-analysis should be interpreted in the light of this recommendation.

Two trials did not provide enough statistical data to be included in the meta-analysis: 

Dalais et al. compared 3 arms of soy, flaxseed and wheat diet. The frequency of hot flashes decreased significantly in wheat diet (p<0.009) and flaxseed ( p<0.09) but not in the soy group (Dalais et al., 1998 ▶). In fact, hot flash decreased by 41% in flaxseed, 51% in wheat diet and 22% in soy groups. No statistical comparison between groups was provided.

**Table 1:** Characteristics of 9 randomized placebo-controlled trials included in our systematic review

**Author,** **Year**	**Menopausal status**	**During** **(wk)**	**Age**	**Drop out%**	**Number patient**	**Intervention**	**Randomization technique**	**Blinding method**	**Intention-to treat reporting**	**Side effect**
**Colli , 2012**	Parallel	24wk	Flaxseed extract /53 Flaxseed meal /54 Placebo/56	17%	Flaxseed extract/28 Flaxseed meal/22 Con/25	Flaxseed extract (containing 100 mg Lignans) Flaxseed meal (containing 270 mg of Lignans) Con= 1000 mg of collagen	No	Unclear	No	No serious
**Pruthi, 2012**	Parallel	6WK	Flaxseed/53 Control/53	18%	Flaxseed/69 Con/77	Flaxseed bar( containing 410 mg of lignans Con/ placebo	Yes	Unclear	No	Flaxseed experience more diarrhea placebo experience more bloating
**Simbalista, 2010**	Parallel	12 WK	Trea=52 Con=52	3%	Treatment=20 Con=18	Bread containing 25 g of flaxseed (46 mg lignans) Con /wheat bran (,1 mg lignans; control)	Yes	Double blind	No	Both group experience intestinal flow, flatulence
**Lewis, 2006**	Parallel	16WK	Soy/53 Flaxseed/53 Placebo/52	11%	Flaxseed /24 Placebo /23 Soy/24	Muffins with 25 g of flaxseed (50 mg of lignans), 25 g soy (42 mg of isoflavones), Con/ wheat	Yes	Double blind	Yes	--------------
**Dodin, 2005**	Parallel	51 l	Flaxssed/54 Calcium /55	105%	Flaxseed Control/ wheat	Flaxssed= 196ug total lignans Control= wheat	No	Double blind	No	-------
**Dalais** **.** ** 1998**	Cross over	2 X 12 weeks with 4 week washout period	Soy/53 Flaxseed= 54 Wheat/53	15%	Soy/44 Flaxseed/44 Wheat/44	Soy/45 mg Flaxseed/45mg Wheat/45 mg	No	Unclear	No	-------------
**Ghazanfarpour, 2013**	Parallel	12 WK	Hypericum perforatum/53 Vitex agnus-castus/52	18%	Hypericum perforatum/31 Vitex agnus-castus/33	Hypericum perforatum/31 Vitex agnus-castus/33	No	Unclear	No	Hypericum perforatum/ Headache and constipation / vitex agnus-castus / headache and Dizziness in the
**Chung,** **2007**	Parallel	8WK	Cimicifugae racemosa rhizome +Hypericum perforatum Control/placebo/51.02 P/50.43	13%	Treatment/42 Placebo /35	Treatmen group/.0364 ml of extract from Cimicifugae racemosa rhizome plus84 mg of dried extract from Hypericum perforatum Control/placebo	No	Unclear	No	In placebo group Gastro­ intestinal complaints/4 Generalized Ache n/1 Weight gain n/1
**Diana van, 2009** **Australia**	Parallel	8WK	T/52.5 P/51.9	7%	T/47 Placebo /46	Hypericum perforatum (contain 990 Kg of hypericin)+ V. Agnus-castus tablet( contained extract equivalent to500 mg of dry fruit),	No	Double blind	No	Two on isoflavones And one on placebo) had a discreet vaginal bleeding During the study

Another study by Pruthi et al. showed no significant difference between flaxseed and placebo groups regarding decrease in percentage change of hot flashes. There was a comparable decrease of 29% for flaxseed group and 28% for control group, which was not statistically significant (p=0.90) (Pruthi et al., 2012 ▶). 

To sum up, flaxseed had better effect as compared to placebo on decreasing the frequency of hot flashes, although the difference was not statistically significant.

**Comparison of the Flaxseed vs. Soy**

Two trials (Dalais et al., 1998 ▶; Lewis et al., 2006b ▶) compared the effects of flaxseed and soy regarding hot flash frequency. Dalais et al. trial compared 3 arms: soy, flaxseed and wheat diet (Dalais et al., 1998 ▶). Hot flashes frequency showed a significant decrease of 41% in the flaxseed and a non-significant decrease of 22% in the soy group. No statistical comparison between groups was provided. Another study by Lewis et al. compared 3 arms: soy, flaxseed and placebo (Lewis et al., 2006b ▶). Comparison of all groups did not show any significant difference. In fact, hot flash frequency decreased from 3.06 to 2.88 (-0.5%) in soy group, from 3.30 to 2.90 (-12%) in the placebo group and from 2.89 to 2.35 (-19%) in the flaxseed group. 

To sum up, flaxseed seems to be more effective as compared to soy and placebo in decreasing the frequency of hot flashes (Dalais et al., 1998 ▶) .

**The effect of flaxseed on n** **ight sweating**

Dodin et al. assessed the effect of flaxseed on night sweating (Dodin et al., 2005b ▶). Night sweats decreased more efficiently in the flaxseed group as compared to the placebo (-1.11 ±1.48 vs. -0.89 ± 1.20), which was not statistically different.

**Hormonal effects of flaxseed**

*The effect of flaxseed on the follicle stimulating hormone (FSH) levels*

**Figure.4:**
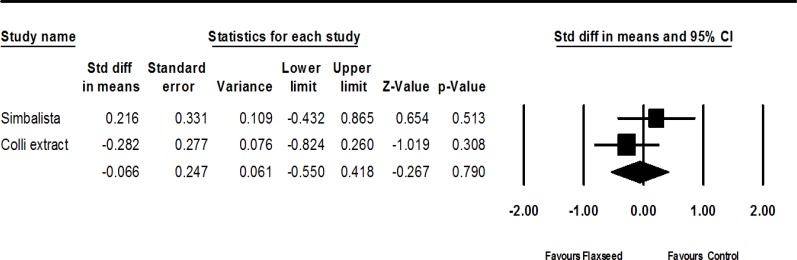
Effects of flaxseed on FSH, The horizontal lines denote the 95% CI, ■ point estimate (size of the square corresponds to its weight); ♦, combined overall effect of treatment

 Two trials (Colli et al., 2012 ▶; Simbalista et al., 2010 ▶) provided complete statistical data to be included in the meta-analysis. Standardized difference in means of FSH demonstrated greater decrease in flaxseed group as compared to placebo (although not statistically significant) (SMD-0.066; 95% CI: -0.550 to 0.418 p=0.790; heterogeneity p=0.248; I^2^=25%; random effects model). The forest plot is shown in Figure 4.

*The effect of flaxseed on the estradiol levels*

**Figure 5:**
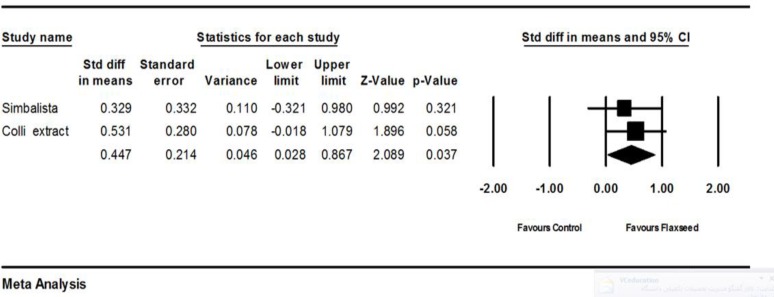
Effects of flaxseed on estradiol levels, the horizontal lines denote the 95% CI, ■ point estimate (size of the square corresponds to its weight); ♦, combined overall effect of treatment

 Two trials (Simbalista et al., 2010 ▶; Colli et al., 2012 ▶) provided complete statistical data to be included in the meta-analysis. Standardized difference in means of estradiol change showed more increase in flaxseed group as compared to placebo (SMD 0.447 95% CI: 0.028 to 0.867 P=0.037; heterogeneity P=0.643; I^2^=0%; 90 women random effects model). Overall, based on two trials, flaxseed showed an estrogenic effect. Further trials are needed to confirm this finding. The forest plot is shown in Figure 5. 

**The effect of flaxseed on endometrial thickness**

**Figure 6:**
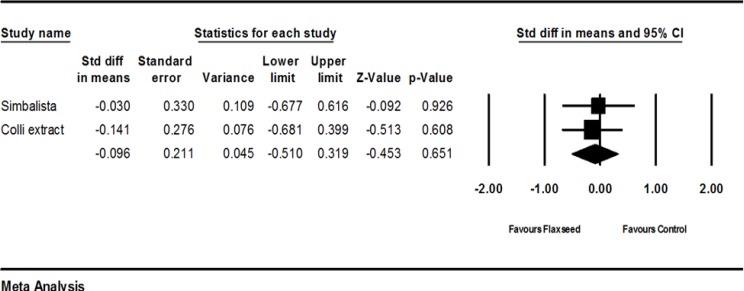
Effects of red clover on endometrial thickness, the horizontal lines denote the 95% CI, ■ point estimate (size of the square corresponds to its weight); ♦, combined overall effect of treatment

 Two trials (Simbalista et al., 2010 ▶; Colli et al., 2012 ▶) provided complete statistical data to be included in the meta-analysis. Flaxseed group showed greater decrease regarding endometrial thickness as compared to placebo (SMD -0.096; 95% CI: -0.510 to 0.319; p=0.651; heterogeneity p=0.248; I^2^=25%; random effects model). The forest plot is shown in Figure 6. To sum up, flaxseed led to slight decrease in endometrial thickness as compared to the control group. Further trials are needed to confirm this finding. 

**The effect of flaxseed on the maturation value**

Two trials (Colli et al., 2012 ▶; Dalais et al., 1998 ▶) assessed the effect of flaxseed on maturation value. One trial by Coli et al. compared 3 arms (1 g of flaxseed extract containing 100 mg of lignan, 90 g of flaxseed meal containing 270 mg of lignan and placebo) (Colli et al., 2012 ▶). Unexpectedly, both flaxseed extract and meal groups showed a reverse effect on maturation value. Maturation of vaginal epithelium decreased from 39.8 ±15.1 to 32.4 ± 18.3 (-18%) in the flaxseed extract group, and from 41.6 ±10.9 to 36.9 ± 16.0 (%11) in the flaxseed meal group and from 35.7 ±12.9 to 32.0 ± 19.0 (%10) in the placebo group. Each of intervention groups of flaxseed extract and flaxseed meal showed greater decrease regarding maturation of vaginal epithelium as compared to the placebo; however, difference between each of the interventions and placebo was not statistically significant.

Another trial by Dalais et al. compared 3 arms: soy, linseed diets (flaxseed) and wheat diet. Maturation value increased by 5.5% in the linseed diets and by 11% in the wheat diet but in the soy group the increase was statistically significant (103%, <0.05) (Dalais et al., 1998 ▶).

***Vitex agnus-castus vs.*** ***H. perforatum***

One trial by Ghazanfarpour et al. compared *Vitex agnus-castus* and H. perforatum. Baseline severity means of hot flashes were 3.67 ± 0.74 for St. John’s Wort and 3.58 ± 0.84 for *Vitex agnus-castus* group (Ghazanfarpour et al., 2013). There was a comparable decrease of John’s Wort group John’s Wort (-61%) and in Vitex agnus-castusgroup (-57%) hot flashes, which was not statistically significant (P=0.68).

**Black cohosh and** ***H. perforatum*** **combination vs. placebo**

Chung et al. compared black cohosh and H. perforatum combination with placebo and found a gradual decrease in hot flash intensity in the intervention group (7.52, 3.24 and 1.97) and in control group score (7.43, 6.29 and 3.54) at three time points: baseline, 4, and 8 weeks (Chung et al., 2007 ▶). Comparison between groups showed a statistically significant difference at 4 (p=0.04) and 8 (p=0.02) weeks.

***Vitex agnus-castus*** **and** ***H. perforatum*** **combination** **vs. placebo**

One trial by Diana van Die et al. compared *Vitex agnus-castus* and H. perforatum combination with placebo (van Die et al., 2009 ▶). After 16 weeks, patients in both groups experienced a statistically significant reduction in hot flashes (p<0.0001). In fact, daily flashes

scores decreased from 16.35 ±1.30 to 9.32±1.36 (42%) in placebo group and from 16.53 ±1.32 to 10.86 ±1.34 (34%) in intervention group. Comparison between groups did not show statistically significant differences (p=0.42). 

**The effect of flaxseed on vasomotor symptoms**

Our results revealed that flaxseed consumption decreased vasomotor symptoms (hot flash frequency and intensity and night sweats) more efficiently as compared to the control group, which was not statistically significant. Our result is consistent with another systematic review which showed beneficial effect of flaxseed on hot flashes, without any statistical significance (Dew and Williamson, 2013 ▶).

**Maturation value**

Two trials assessed the effect of flaxseed on maturation value (Colli et al., 2012 ▶; Dalais et al., 1998 ▶). One gram of flaxseed extract containing 100 mg lignan and 90 g flaxseed meal containing contain 270 mg lignan cannot prevent progressive post-menopausal changes of vaginal epithelial cell (Colli et al., 2012 ▶) while 45 mg flaxseed (containing unknown levels of isoflavone) slightly increased the maturation value by 5% (Dalais et al., 1998 ▶). Based on the current result, no consistent conclusion can be made in this regard and further trials are still needed.

**Risk of breast cancer**

**Cancer-promoting effects**

 Current systematic review showed that flaxseed may increase the risk of breast cancer. Meta-analysis of the included data showed that the mean increase in estradiol level was higher in the flaxseed group as compared to the placebo. A meta-analysis including a few prospective studies concluded that increase in circulating estradiol concentration was associated with the increased risk of cancer breast (Hooper et al., 2009 ▶). 

**Cancer-protecting effects**

On the other hand, there is evidence for protective effect of flaxseed on breast cancer. A recent study by Lowcock et al. showed that ingestion of flaxseed with high concentrations of lignans, was associated with a reduced risk of breast cancer (Lowcock et al., 2013 ▶). Another meta-analysis of epidemiological studies concluded that consumption of plants containing lignans was associated with a reduced risk of breast cancer only in postmenopausal period (Buck et al., 2010 ▶). 

 Another evidence for protective effect for flaxseed is a slight decrease in endometrial thickness (although non-significant) in flaxseed group as compared with the control group. Based on the current result, we need more compelling and larger trials to judge more accurately regarding promoting or protective effect of flaxseed on breast cancer.

**The effect of the black cohosh and** ***H. perforatum*** **combination on hot flash intensity**

Chung et al. showed significant beneficial effect of black and H. perforatum combination as compared to the control group on the hot flashes (Chung et al., 2007 ▶). One prospective controlled open-label observational study by Briese et al. which was not included in the current systematic review (we only included randomized controlled trials) compared the black cohosh and *H. perforatum* combination with black cohosh alone (Briese et al., 2007 ▶). Black cohosh and *H. perforatum* combination had higher efficacy compared to black cohosh alone regarding hot flashes reduction. However, larger trials are still needed to investigate the synergistic effect of black cohosh and *H. perforatum*.

**Limitation**

Suboptimal methodological quality of the included trials in the current systematic review is the main limitation of our study. Almost none of the included trials reported randomization technique, blinding assessment and intention-to treat reporting. Further trials should take into consideration the CONSORT checklist to improve methodological quality as well as their reporting.

## Conclusion

Our systematic review showed beneficial effect of flaxseed on vasomotor symptoms of post-menopausal women. However no consistent conclusion can be reached regarding estrogen dependent cancer and vaginal maturation value. Further high quality trials are still needed to address these issues. 

## Conflict of interest

The authors report no conflicts of interest. The authors alone are responsible for the content and writing of the paper.
